# Intrinsic TGF-β2-triggered SDF-1-CXCR4 signaling axis is crucial for drug resistance and a slow-cycling state in bone marrow-disseminated tumor cells

**DOI:** 10.18632/oncotarget.2826

**Published:** 2014-11-25

**Authors:** Takuya Nakamura, Satoru Shinriki, Hirofumi Jono, Jianying Guo, Mitsuharu Ueda, Mitsuhiro Hayashi, Satoshi Yamashita, Andries Zijlstra, Hideki Nakayama, Akimitsu Hiraki, Masanori Shinohara, Yukio Ando

**Affiliations:** ^1^ Department of Oral and Maxillofacial Surgery, Graduate School of Medical Sciences, Kumamoto University, Kumamoto, Japan; ^2^ Department of Laboratory Medicine, Graduate School of Medical Sciences, Kumamoto University, Kumamoto, Japan; ^3^ Department of Clinical Pharmaceutical Sciences, Graduate School of Pharmaceutical Sciences, Kumamoto University and Department of Pharmacy, Kumamoto University Hospital, Kumamoto, Japan; ^4^ Department of Neurology, Graduate School of Medical Sciences, Kumamoto University, Kumamoto, Japan; ^5^ Department of Breast and Endocrine Surgery, Graduate School of Medical Sciences, Kumamoto University, Kumamoto, Japan; ^6^ Department of Pathology, Microbiology, and Immunology, Vanderbilt University Medical Center, Nashville, TN, USA

**Keywords:** bone marrow, disseminated tumor cells, drug resistance, slow-cycling cells, TGF-β2, SDF-1-CXCR4 axis

## Abstract

Dormant or slow-cycling disseminated tumor cells (DTCs) in bone marrow (BM) are resistant to conventional therapy in various cancers including head and neck squamous cell carcinoma (HNSCC), although the molecular mechanisms remain largely unknown. This study aimed to identify the intrinsic molecular mechanisms underlying drug resistance in BM-DTCs. We used *in vivo* selection of the human HNSCC cell line HEp3, which mimics non-proliferative BM-DTCs in mice, to establish BM-DTC-derived (BM-HEp3) and lung metastases-derived (Lu-HEp3) sublines. Both sublines had higher migration activity and shortened survival in a murine xenograft model compared with parental (P-HEp3) cells. Slow-cycling BM-HEp3 cells had intrinsically enhanced cisplatin resistance compared with Lu-HEp3 cells, which also manifested this resistance but proliferated rapidly. The drug resistance and slow-cycling state of BM-HEp3 cells depended on enhanced positive feedback of the signaling axis of stromal cell-derived factor-1 (SDF-1)-C-X-C chemokine receptor-4 (CXCR4) via their overexpression. Interestingly, BM-DTCs highly expressed transforming growth factor-beta 2 (TGF-β2) to maintain SDF-1-CXCR4 overexpression. Inhibition of SDF-1-CXCR4 signaling by down-regulating TGF-β2 fully reversed the drug resistance of BM-HEp3 cells via reactivation of cell proliferation. These data suggest that the intrinsic TGF-β2-triggered SDF-1-CXCR4 signaling axis is crucial for drug resistance dependent on a slow-cycling state in BM-DTCs.

## INTRODUCTION

Minimal residual disease caused by solitary disseminated tumor cells (DTCs) is often observed in bone marrow (BM) in patients with different types of cancer [1]. Although most DTCs in BM aspirates are negative for proliferation markers [1,2], the abundance of these cells at the time of surgery or after treatment directly correlates with reduced metastasis-free survival, even for cancers in which overt skeletal metastases, such as head and neck squamous cell carcinoma (HNSCC), are rare [1,3,4]. These findings suggest that BM-DTCs eventually leave dormancy, which is functionally defined by quiescence, to initiate metastasis [5]. Also, the BM can be the source for dissemination into other organs [6]. One most important obstacle to be overcome in cancer therapy is resistance of BM-DTCs to conventional chemotherapeutic agents [7,8]. Their drug resistance may result from coordinated growth arrest and a survival scheme that allow long-term dormancy [9]. Understanding how these cells resist conventional therapy and persist in a viable state for prolonged periods is of fundamental clinical interest.

Chemotherapeutic drug resistance in BM-DTCs may arise from interactions between cell-intrinsic and environment-mediated mechanisms [7,8]. Indeed, components in the BM environment can protect DTCs from chemotherapeutic agents by similar mechanisms [7]. However, whether such *de novo* mechanisms are sufficient for BM-DTC resistance, i.e., whether any cancer cells can become dormant and lead to residual disease only if they are in the BM microenvironment, remains unknown. Recent studies on organ-specific metastatic traits revealed that only a small population of cancer cells with a unique survival mechanism can survive in the BM or lung [10,11] and that DTCs in each organ (e.g., lung, liver, and BM) have distinct, intrinsic molecular characteristics [12]. Moreover, the likelihood of metastasis to certain organs may be predicted from gene expression patterns of primary tumors [13-15]. These findings suggest the presence of intrinsic resistance mechanisms in DTCs or metastatic cells that may be preselected in primary tumors and that differ in terms of the organs where they lodge (e.g., lung vs. BM). However, whether intrinsic properties are involved in drug resistance in DTCs in the BM or other sites is yet unknown because of the lack of studies on DTCs themselves. This issue may have implications for the general question of whether DTCs or metastatic cells in various sites respond similarly to the same therapies.

*In vivo* selection is effective in differentiating highly disseminating or metastatic subpopulations from an original cell mixture, more effective, in fact, than direct analyses of cancer cell populations that were established from patients and that are likely heterogeneous, with different genomic characteristics and abilities to metastasize to distant secondary sites [12,16,17]. The human HNSCC cell line HEp3 produces overt spontaneous metastasis in multiple organs, such as lung and lymph nodes in murine and avian systems, and it mimics metastasis in patients with HNSCCs [4]. This model has non-proliferative DTCs in the BM, as observed in HNSCCs and other malignancies [2,18]. In the present study, we utilized the HEp3 system to identify intrinsic molecular mechanisms underlying drug resistance in BM-DTCs, which may induce BM-DTCs to remain dormant for long-term periods. To achieve this goal, we compared the phenotypic and molecular characteristics of a BM-derived subpopulation with not only the parental population but also lung-derived metastatic cells as another aggressive population.

## RESULTS

### Aggressive Phenotypic Features of BM-Derived DTCs

The HNSCC cell line HEp3 forms metastases in multiple organs such as lungs, lymph nodes, liver, and spleen in mice and in avian systems [18,20]. These cells are known to not develop bone metastases, at least in the same time frame as for development of spontaneous metastases in lungs and lymph nodes, and this model mimics the behavior of non-proliferative DTCs in the BM in patients with HNSCCs [1,18,21].

**Figure 1:**
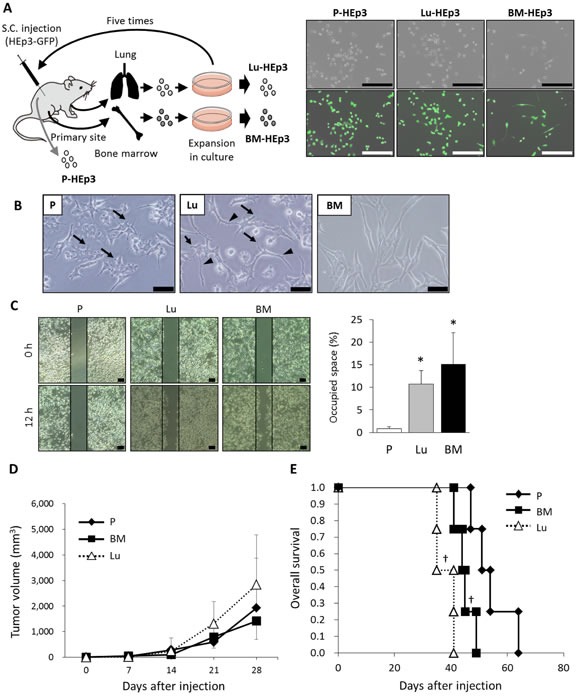
Phenotypes of BM-derived DTCs (A) Schematic representation of the procedure used for *in vivo* selection (left panel). HEp3 cells expressing green fluorescent protein (GFP) (5 × 10^6^) were injected subcutaneously (S.C.) into mice. At 30-40 days after injection, HEp3 cells in the primary site (P-HEp3) and DTCs in lung and BM were isolated and then expanded in monolayer culture. After subconfluent growth, lung- and BM-derived cells were injected subcutaneously into mice again. These transplantations were repeated five times, and the resultant sublines derived from BM and lung were called BM-HEp3 and Lu-HEp3, respectively. Phase-contrast and corresponding images merged with GFP fluorescence for P-HEp3, Lu-HEp3, and BM-HEp3 cells are shown (right panels). Scale bars indicate 400 μm. (B) Representative images of cell morphology of the P-HEp3 (P), Lu-HEp3 (Lu), and BM-HEp3 (BM) sublines. Scale bars indicate 50 μm. Arrows and arrowheads indicate filopodia-like and dendrite- or axon-like protrusions, respectively. (C) The HEp3 sublines were wounded by scratching and were then incubated in serum-free medium for 12 hours. Cell migration into the wound area was visualized with a phase-contrast microscope and photographed. Representative photographs are shown (left panels), and the quantitative results provide the means ± SEM of triplicate samples (right panel). ^*^P < .01. (D) Tumor growth after the HEp3 sublines were injected subcutaneously into mice. The graph shows mean tumor growth rates ± SD for four animals per experimental condition. (E) Kaplan-Meier plots of overall survival of each experimental group. ^†^*P* < .05 (log-rank test).

To clarify the mechanism underlying chemotherapeutic drug resistance in dormant or slow-cycling DTCs in the BM, we established BM- and lung-derived DTC sublines (Figure 1A). We injected HEp3 cells expressing green fluorescent protein subcutaneously into mice. After 4-5 weeks, we isolated HEp3 cells from the injection site, which we designated the parental line P-HEp3, and DTCs from the BM and the lung metastases. We expanded these two groups of DTCs in culture and then reinjected them into mice. We repeated this *in vivo* transplantation five times. Isolated DTCs from the BM and the lung metastases after the fifth transplantation were named BM-HEp3 and Lu-HEp3, respectively (Figure 1A, left panel). GFP expression of P-HEp3, Lu-HEp3, and BM-HEp3 cells was confirmed (Figure 1A, right panels). Consistent with previous reports [1,18,20], although overt metastases were observed in the lung at 5 weeks at the latest after injection, visible skeletal metastases did not occur throughout the five transplantations (data not shown). We analyzed the phenotypic characteristics of these BM- and lung-derived sublines and compared them with those of P-HEp3.

One of the obviously different characteristics in the HEp3 sublines was cell morphology (Figure 1B). Almost all P-HEp3 cells had a star shape with many filopodia-like protrusions. The shape of the Lu-HEp3 cells mostly resembled that of the P-HEp3 cells but had longer dendrite- or axon-like protrusions. The BM-HEp3 cells looked quite different from the parental and Lu-HEp3 cells: they were larger and had a fibroblastic appearance, with few or no protrusions. These data indicated that BM-HEp3 and Lu-HEp3 cells were distinct populations even though they both originated from a single cell line. To evaluate their functional differences, we performed the wound-healing assay (Figure 1C). Lu-HEp3 and BM-HEp3 cells had markedly enhanced motility compared with the parental line, a characteristic that supported their high disseminating capacity.

To confirm this potential aggressive dissemination, we injected mice subcutaneously with cells from different lines. Although no significant difference in tumor growth rate occurred (Figure 1D), the survival times of mice bearing BM-HEp3 or Lu-HEp3 tumors were significantly shorter than that of mice with P-HEp3 cells (Figure 1E). Together, these data indicate that both Lu- and BM-derived subpopulations had more aggressive characteristics compared with their parental population, at least with regard to disseminating ability.

### BM-DTCs Are Slow-Cycling Cells and Are Resistant to Anticancer Drugs

**Figure 2:**
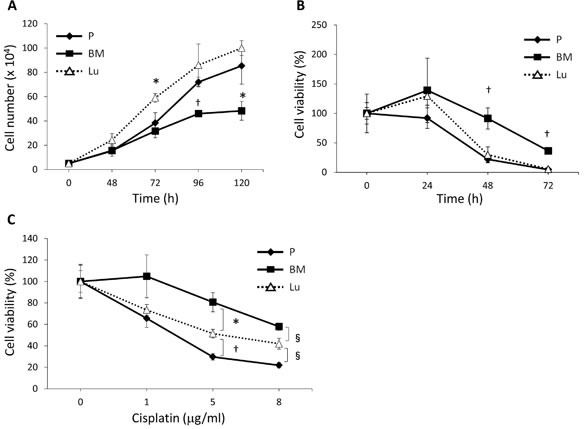
Cell proliferation and cisplatin sensitivity of BM-derived and lung-derived DTCs (A) Proliferation rate of P-HEp3 (P), Lu-HEp3 (Lu), and BM-HEp3 (BM) cells. The number of cells in each subline was determined at the indicated time points after plating. ^*^P < .05, ^†^*P* < .01 compared with P-HEp3 cells. (B) Cells were serum-starved and then their survival was evaluated at the indicated time points. ^†^*P* < .01 compared with P-HEp3 and Lu-HEp3 cells. (C) Cells were treated with cisplatin at the concentrations shown for 48 hours, after which cell numbers were determined. *P < .05, ^†^*P* < .01,^§^*P* < .005. Values are means ± SEM of triplicate samples.

We next investigated the proliferation and survival of each cell line *in vitro*. We found that BM-HEp3 cell proliferation was significantly slower than proliferation of P-HEp3 cells, whereas Lu-HEp3 cells proliferated rapidly (Figure 2A). Although survival of Lu-HEp3 and P-HEp3 cells under serum-free conditions did not appear to differ, the survival rate of BM-HEp3 cells was significantly higher than that of the other cell lines (Figure 2B).

We next assessed the sensitivity of these cell lines to cisplatin, one of the most effective and commonly used chemotherapeutic drugs for HNSCC and many other solid tumors [22]. We found that Lu-HEp3 and BM-HEp3 cells had decreased sensitivity to cisplatin compared with P-HEp3 cells (Figure 2C). BM-HEp3 cells, however, had markedly enhanced resistance compared with Lu-HEp3 cells (Figure 2C), which is consistent with their greater survival ability. These data indicate that BM-DTCs were intrinsically slow-cycling and were resistant to an anticancer drug, with an enhanced survival ability.

### Autocrine Stromal Cell-Derived Factor-1 (SDF-1)- C-X-C chemokine receptor-4 (CXCR4) Axis Contributes to Growth Suppression and Drug Resistance in BM-DTCs

Growth arrest is associated with increased survival and chemoresistance in BM-DTCs [23]. We therefore hypothesized that the slow-cycling state actually observed in BM-HEp3 cells contributes to acquisition of chemoresistance in these cells.

**Figure 3:**
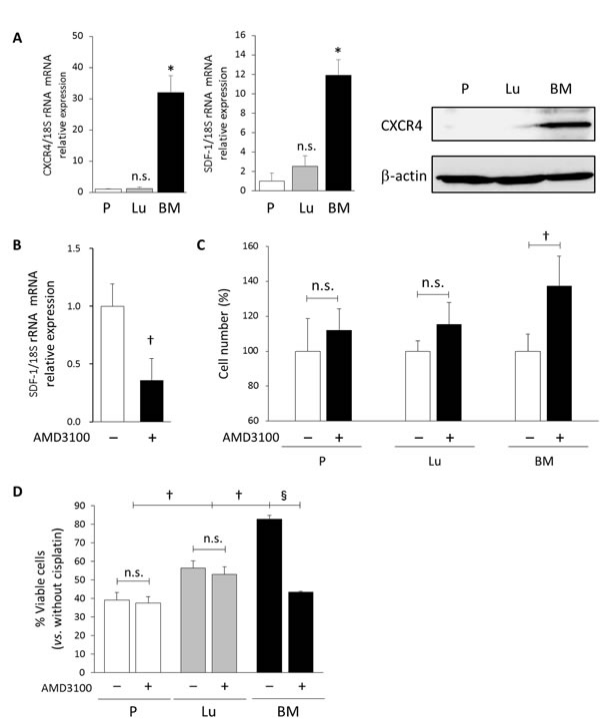
Autocrine SDF-1-CXCR4 signaling maintains a slow-cycling state and drug resistance in BM-derived DTCs (A) mRNA expression of CXCR4 (left) and SDF-1 (middle), and CXCR4 protein expression (right) in the P-HEp3 (P), Lu-HEp3 (Lu), and BM-HEp3 (BM) sublines, cultured in serum-free medium for 24 hours, was determined via qRT-PCR and Western blotting, respectively. ^*^P < .001. n.s., not significant. (B) BM-HEp3 cells were treated with AMD3100 (5 μM) for 24 hours, after which SDF-1 mRNA expression was determined via qRT-PCR. ^†^*P* < .05. (C) The sublines were treated with AMD3100 (5 μM) for 24 hours, after which cell numbers were counted. ^†^*P* < .05. (D) Cells were treated with cisplatin (5 μg/ml) with or without AMD3100 (5 μM) for 48 hours, after which cell numbers were counted. Results are expressed as a percentage relative to cells without cisplatin in each experimental group. ^†^*P* < .05,^§^*P* < .01. n.s., not significant. Values are means ± SEM of triplicate samples.

The signaling mediated by the chemokine SDF-1 (also called CXCL12) and its cognate receptor CXCR4 has a central role in BM homing and is also required for the quiescence and retention of hematopoietic stem cells in the BM [7]. In view of these known functions, we compared the expression of SDF-1 and CXCR4 in BM-HEp3 cells with that in the other cell lines. As Figure 3A illustrates, gene expression of both SDF-1 and CXCR4 in BM-HEp3 cells was much higher than that in the other cell lines. No significant difference in expression of these genes between P-HEp3 and Lu-HEp3 cells was observed. We confirmed increased CXCR4 protein level in BM-HEp3 cells compared to the other cell lines (Figure 3A). These data indicated that SDF-1-CXCR4 was constitutively enhanced in BM-DTCs. Treatment with AMD3100, a CXCR4-specific inhibitor [24], dramatically suppressed SDF-1 transcription in BM-HEp3 cells, which indicated that SDF-1 expression depended an enhanced CXCR4 downstream signal (Figure 3B). Together, these data suggest that BM-DTCs maintained a positive feedback SDF-1-CXCR4 signaling loop.

We next studied whether this SDF-1-CXCR4 signaling was involved in the slow-cycling state and chemoresistance in BM-derived DTCs. We found that inhibition of CXCR4 significantly promoted cell proliferation in BM-HEp3 cells, although no significant change in P-HEp3 and Lu-HEp3 cells occurred (Figure 3C). As a striking result, CXCR4 inhibition led to full reversal of the sensitivity to cisplatin in BM-HEp3 cells, to the same level as that in parental P-HEp3 cells (Figure 3D). Another notable finding was that CXCR4 inhibition did not affect cisplatin sensitivity in the P-HEp3 cells and Lu-HEp3 cells. Taken together, these data suggest that enhanced SDF-1-CXCR4 signaling, likely triggered by CXCR4 overexpression, was required for maintenance of the slow-cycling state and drug resistance in BM-DTCs, which confirmed the close relationship among these phenotypes in BM-DTCs.

### Overexpression of CXCR4 and SDF-1 in BM-DTCs Requires Transforming Growth Factor-Beta 2 (TGF-β2)

**Figure 4:**
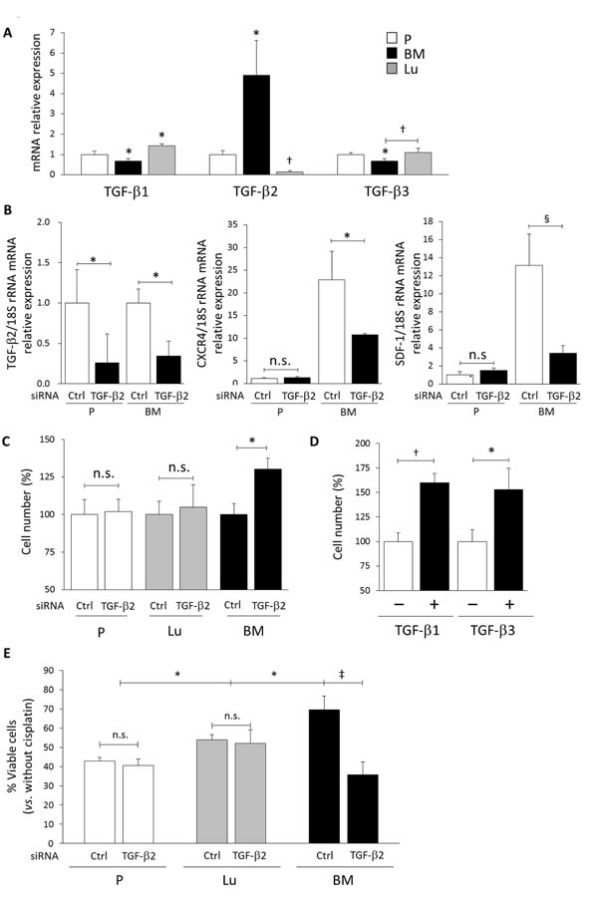
The SDF-1-CXCR4-dependent slow-cycling state and drug resistance in BM-DTCs requires TGF-β2 (A) mRNA expression of TGF-β1, TGF-β2, and TGF-β3 in P-HEp3 (P), Lu-HEp3 (Lu), and BM-HEp3 (BM) cells was determined via qRT-PCR. ^*^P < .05, ^†^*P* < .01 compared with P-HEp3 cells unless otherwise indicated. (B) Expression of TGF-β2 (left), CXCR4 (middle), and SDF-1 (right) mRNA in P-HEp3 and BM-HEp3 cells was measured by means of qRT-PCR 48 hours after TGF-β2 siRNA transfection. ^*^P < .05, ^§^*P* < .005. n.s., not significant. (C) The numbers of P-HEp3, Lu-HEp3, and BM-HEp3 cells were determined 48 hours after transfection with control or TGF-β2 siRNA. ^*^P < .05. (D) BM-HEp3 cells were treated with TGF-β1 (5 ng/ml) or TGF-β3 (5 ng/ml) for 48 hours, and then cell numbers were counted. ^*^P < .05,^†^*P* < .01. (E) Cisplatin (5 μg/ml) was added to P-HEp3, Lu-HEp3, and BM-HEp3 cells at 48 hours after control or TGF-β2 siRNA transfection, followed by incubation for 48 hours in serum-free conditions. Cell numbers were then counted. Results are expressed as a percentage relative to cells without cisplatin in each experimental group. ^*^P < .05, ^‡^*P* < .001. Values are means ± SEM of triplicate samples.

In view of the above findings, we next investigated the mechanisms of CXCR4 overexpression in BM-DTCs. In several types of cancer including HNSCC, TGF-β is a critical regulator of not only hematopoietic stem cell hibernation in the BM [25] but also of metastatic processes, including tumor cell colonization, cell dormancy, and metastatic progression, in distant organs such as bone [18,26-28]. We therefore first assessed expression of all TGF-β isoforms—TGF-β1, TGF-β2, and TGF-β3. Our real-time quantitative reverse transcription-polymerase chain reaction (qRT-PCR) analysis revealed significantly increased TGF-β2 gene expression in BM-HEp3 cells compared with P-HEp3 cells (Figure 4A). In contrast, TGF-β2 expression in Lu-HEp3 cells was much lower than that in P-HEp3 cells. A notable result was that in BM-HEp3 cells, expression of TGF-β1 and TGF-β3 was significantly decreased compared with that in P-HEp3 cells, whereas Lu-HEp3 cells showed increased TGF-β1 expression compared with P-HEp3 cells.

We then investigated whether elevated TGF-β2 expression was involved in the expression of CXCR4 or SDF-1. Indeed, TGF-β2 knockdown via siRNA led to a dramatic decrease in expression of both CXCR4 and SDF-1 in BM-HEp3 cells, but P-HEp3 cells showed no apparent change in CXCR4 and SDF-1 expression (Figure 4B). Of note, TGF-β2 knockdown significantly increased expression of both genes in Lu-HEp3 cells (Supplementary Figure S1). CXCR4 inhibition had no apparent effect on TGF-β2 transcription in BM-HEp3 cells (Supplementary Figure S2). These results indicated that an enhanced SDF-1-CXCR4 signaling axis in BM-DTCs depended strongly on the TGF-β2 signal in a cell-autonomous fashion.

**Figure 5:**
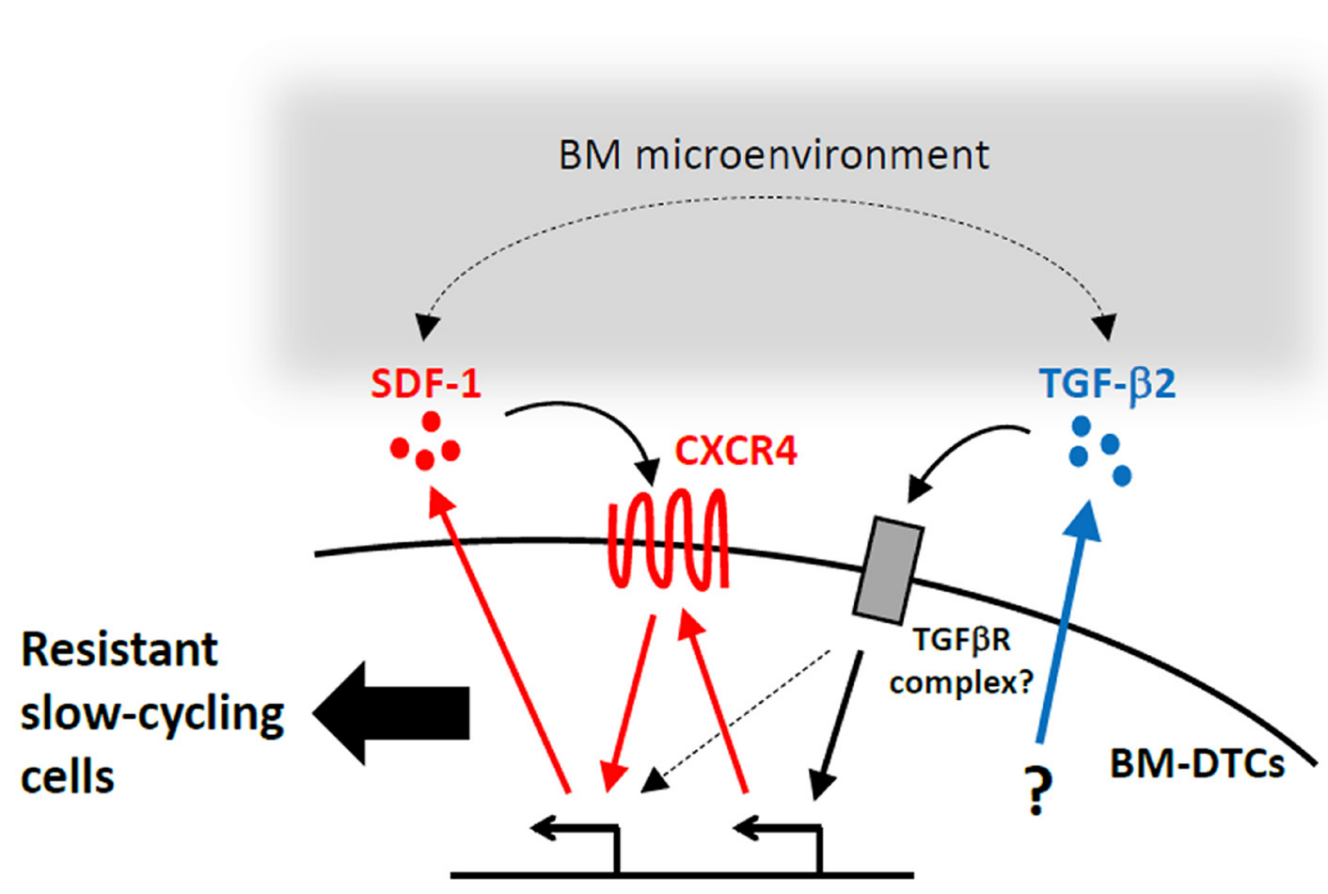
A proposed mechanism of drug resistance and the slow-cycling state in BM-DTCs This scheme summarizes data from this and other studies and presents a model illustrating how cell-autonomous and BM microenvironment-mediated mechanisms may synergistically contribute to drug resistance and a slow-cycling state in DTCs. As demonstrated in our study, BM-DTCs overexpress TGF-β2 through a yet unknown mechanism. This cytokine maintains expression of CXCR4 and SDF-1, which results in drug resistance and a slow-cycling state in a cell-autonomous fashion. SDF-1 and TGF-β2 in the BM microenvironment may facilitate these signaling pathways, which may contribute to creating conditions that would allow DTCs to persist as dormant residual disease.

In addition, knockdown of TGF-β2 expression stimulated proliferation of only BM-HEp3 cells, in a similar manner as did CXCR4 inhibition (Figure 4C). Addition of TGF-β1 or TGF-β3 significantly increased proliferation of these cells (Figure 4D). Moreover, we found that knockdown of TGF-β2 completely abolished cisplatin resistance in BM-HEp3 cells, but we did not note any change in cisplatin sensitivity in P-HEp3 and Lu-HEp3 cells, which we did observe when CXCR4 was inhibited (Figure 4E). Taken together, our data demonstrate that TGF-β2 overexpression was responsible for an enhanced SDF-1-CXCR4 signaling axis and for the subsequent chemoresistance and slow-cycling state in BM-derived DTCs (Figure 5).

## DISCUSSION

Dormant or slow-cycling DTCs, which often occur in the BM, are resistant to conventional therapy and are thought to be a cause of relapse and metastasis in cancers. However, the molecular mechanisms responsible for the drug resistance of these cells are still poorly understood. Using an *in vivo* selection approach, we demonstrated here that in BM-derived DTCs, but not in the primary cell population or in a lung metastatic derivative, autocrine TGF-β2 maintained the drug resistance and a slow-cycling state via enhancement of the SDF-1-CXCR4 signaling axis.

Our data showed that the drug-resistant phenotype of the BM-derived DTCs (BM-HEp3 cells) was closely linked to the slow-cycling state, whereas lung-derived metastatic cells (Lu-HEp3 cells) manifested a different modality. Slow-cycling tumor cells are known to be more drug-resistant than other tumor cells, although direct proof of this is lacking [23]. Indeed, this coordinated growth arrest and survival program would favor long-term persistence of DTCs in the BM microenvironment, as clinically observed. Our evidence suggests that DTCs lodged in the BM elicit this trait themselves, through an intrinsic mechanism: a positive feedback SDF-1-CXCR4 signaling loop, which is a frequently observed autocrine system [29] and, in this case, is likely to be initiated by CXCR4 overexpression. The SDF-1-CXCR4 axis has reportedly promoted drug resistance through several signaling pathways including activated focal adhesion kinase, extracellular signal-related kinase, and Akt [7]. Our data suggest that such a protective effect is dominant in BM-DTCs and that this major effect may result from high expression levels of SDF-1 and CXCR4. SDF-1 is a chemokine that is abundant in the BM. Thus, SDF-1-CXCR4 axis-mediated homing of tumor cells to the BM is commonly observed in various hematopoietic malignancies [30] and carcinomas including HNSCC [6,31,32], whereas inhibition of CXCR4 blocks this homing [7]. This signaling axis is also indispensable for quiescence and retention of hematopoietic stem cells in the BM [7,33]. Furthermore, inhibition of CXCR4 by AMD3100 mobilizes tumor cells out of the BM and leads to increased chemosensitivity, although the effect of this treatment on the cell cycle remains to be determined [7]. Taken together, our data suggest that CXCR4 overexpression, which can trigger SDF-1 expression, and the corresponding drug resistance and/or slow proliferation are common characteristics of DTCs in the BM, even though the mechanism of CXCR4 overexpression is still largely unexplained.

In our study here, we identified TGF-β2, which is also highly expressed in BM-DTCs, as an essential and sufficient factor for maintaining high CXCR4/SDF-1 expression and drug resistance and a slow-cycling state in BM-DTCs. Recent studies showed that TGF-β2 and TGF-β1 can induce CXCR4 expression in several types of tumor cells including HNSCC cells [34-37] and leukocytes [38-40], via TGF-β type I receptor-dependent non-Smad signaling pathways. Functionally, TGF-β2 is an established inducer of epithelial and mesenchymal transition, an important process for tumor cell dissemination [41]. Epithelial and mesenchymal transition increases cell survival and drug resistance [42]. Notably, TGF-β-induced epithelial and mesenchymal transition and survival require induction of CXCR4 expression [35,43]. Our data (Figure 4B and Supplementary Figure S1) indicate the involvement of unique or dominant signaling pathways in BM-DTCs. Although TGF-β isoforms share several signaling pathways [41], the roles of these isoforms in cell proliferation, especially metastatic tumor growth, are different. Our evidence (Figure 4A and D) suggests that the slow-cycling state of BM-DTCs is maintained by not only enhanced TGF-β2 expression but also down-regulation of TGF-β1 and TGF-β3. In fact, TGF-β2 functions as a growth suppressor in normal and cancer cells [18,25,44]. However, TGF-β1 induces prometastatic growth of DTCs in bone and lung [26-28]. In addition, TGF-β1 and TGF-β3, but not TGF-β2, are abundant in vigorous metastases in bones of patients with breast cancer [10]. Thus, the differential expression pattern of each TGF-β isoform in slow-cycling BM-HEp3 cells may reflect unique roles for these proteins in metastatic progression. Additional investigations to elucidate molecular mechanisms regulating TGF-β isoform expression and CXCR4 signaling may give novel insights into the coordinated growth arrest and survival program of BM-DTCs.

As recent studies demonstrated [45], the behavior of DTCs in certain organs results from interaction of DTCs and their microenvironments. An important finding was the abundance of both TGF-β2 and SDF-1 in the BM microenvironment of humans and mice [18,46,47]. This result suggests synergism of TGF-β2, SDF-1, and CXCR4 in tumor cells and the BM microenvironment, as observed in other sites [29,38,48]. Thus, we propose that this enhancing mechanism enables the quiescent BM-DTC phenotype with increased drug resistance to persist as dormant residual disease (Figure 5). This proposal is supported by a recent study in which BM microenvironment-derived TGF-β2 was required for BM-DTC quiescence in this HEp3-HNSCC model and a breast cancer model [18]. An interesting possibility is that specific cells in the primary tumor cell population that can easily adapt to the microenvironments where the cells will lodge may be preselected for metastatic tropism [46,49]. In this regard, it should be noted that BM-HEp3 tumor led to shorter mouse survival than P-HEp3 even though apparent skeletal metastases did not occur, which suggest that BM-Hep3 subpopulation also have propensity for dissemination to distant organs (e.g., lung) other than BM. Additional investigations may provide evidence to support this hypothesis if intrinsic mechanisms of drug resistance in DTCs in each organ also are consistent with the “seed and soil” theory of metastasis that was proposed to explain the metastatic preference of certain cancer cells for specific organs [50].

In conclusion, we used the HNSCC model to demonstrate here, for the first time, that the autocrine TGF-β2-SDF-1-CXCR4 signaling axis is crucial for drug resistance and the slow-cycling state in BM-DTCs. Inhibition of CXCR4 or TGF-β2 may be a promising strategy to overcome this drug resistance in BM-DTCs and prevent HNSCC recurrence. Our data emphasize the importance of understanding cell-autonomous mechanisms underlying drug resistance in DTCs and metastatic cells, mechanisms that may differ in organs where such cells lodge (e.g., primary tumor, lung, and BM), as related to microenvironment-derived protective functions.

## METHODS

### Cell Lines and Cell Culture

HEp3 cells expressing green fluorescent protein (HEp3) were kindly provided by Dr. Zijlstra. HEp3 cells were originally derived from a lymph node metastasis of a patient with HNSCC [19]. We established the lung metastases-derived and BM-DTC-derived cell lines from lung and BM preparations, respectively (see below). Cells were grown in Dulbecco's modified Eagle's medium (Gibco, Carlsbad, CA, USA) with 10% heat-inactivated fetal bovine serum (Gibco) in a humidified 5% CO_2_ incubator at 37°C unless otherwise stated.

### Establishment of BM- and Lung-Derived Cell Lines

Crlj:SHO-*Prkdc^scid^ Hr^hr^* mice, 4-6 weeks old, were purchased from Charles River Japan (Yokohama, Japan) and maintained at the Center for Animal Resources and Development of Kumamoto University. The mice were handled according to the animal care policy of Kumamoto University. HEp3 cells were harvested and resuspended in PBS, after which 5 × 10^6^ cells were injected subcutaneously into the left axilla of each mouse. The mice were killed 30-40 days after injection, and a necropsy was performed immediately. The thigh bone was separated from the body at the joints. After skin and muscle were removed with a scalpel, both ends of the long bone were cut open. A 20-ml syringe with a 26-gauge needle was filled with 10 ml of PBS and inserted into one end of the long bone. Applying pressure to the syringe forced out the BM cells and tumor cells from the other end. Lungs were excised from the mice and minced in PBS containing DISPASE II (2.5 mg/ml; Wako Pure Chemical Industries, Osaka, Japan). Each BM and lung suspension was seeded in Dulbecco's modified Eagle's medium with 10% fetal bovine serum, 100 U/ml penicillin, and 100 μg/ml streptomycin (Gibco). After subconfluent growth was observed, lung- and BM-derived tumor cells were injected subcutaneously into mice again. These *in vivo* transplantations were repeated five times. Tumor cell lines derived from the BM and lung after the fifth transplantation were designated BM-HEp3 and Lu-HEp3, respectively. In addition, HEp3 cells isolated from the injection site after the first injection was named P-HEp3, the parental line. Images of cells were taken using an EVOS fl Digital Inverted Fluorescence Microscope (Advanced Microscopy Group, Bothell, WA, USA).

### Tumor Xenograft Generation

For survival experiments, P-HEp3, Lu-HEp3, and BM-HEp3 cells were harvested and resuspended in PBS, after which aliquots containing 5 × 10^6^ cells of the cell lines were injected subcutaneously into the left axilla of each mouse (*n* = 4/group). The health of the mice and evidence of tumor growth were evaluated every 3-4 days. Tumor development was monitored in individual animals by using sequential caliper measurements of length (L) and width (W). Tumor volume was calculated via the formula LW^2^π/6.

### Wound-Healing Assay

Cell migration ability was analyzed by using a wound-healing assay. P-HEp3, Lu-HEp3, and BM-HEp3 cells (9 × 10^4^ cells/ml) were seeded in 12-well plates and incubated at 37°C. Subconfluent monolayer cells were wounded by scratching, after which they were incubated at 37°C in serum-free medium. After 12 hours, cell migration into the wound area was visualized with a phase-contrast microscope and was photographed.

### Cell Proliferation Assay

P-HEp3, Lu-HEp3, and BM-HEp3 cells (9 × 10^4^ cells/well) were seeded in 12-well plates. At the indicated times, viable cells in each well were quantified by using the Luna Automated Cell Counter (Logos Biosystems, Annandale, VA, USA). In some cases, cells were treated with recombinant human TGF-β1, TGF-β3 (10 ng/ml; PeproTech, Rocky Hill, NJ, USA), or the highly selective CXCR4 antagonist AMD3100 (5 μM; Abcam, Cambridge, UK), or cells were transfected with small interfering RNA (siRNA) targeting TGF-β2.

### Transfection with siRNA

P-HEp3, Lu-HEp3, and BM-HEp3 cells were transfected with TGF-β2-specific siRNA by using Lipofectamine 2000 (Invitrogen, Life Technologies, Carlsbad, CA, USA), according to the manufacturer's protocol. Silencer Negative Control (Ctrl) siRNA (Applied Biosystems, Life Technologies, Foster City, CA, USA) was used as the control. The sequences of the TGF-β2-specific siRNA were sense 5′-CUGAACAACGGAUUGAGCUTT-3′ and antisense 5′-AGCUCAAUCCGUUGUUCAGTT-3′ (Sigma, St Louis, MO, USA).

### Drug Sensitivity Assay

P-HEp3, Lu-HEp3, and BM-HEp3 cells (9 × 10^4^ cells/well) were seeded in 12-well or 24-well plates. After 24 hours, various cisplatin concentrations were added to each well, the cells were incubated at 37°C for another 48 hours, and viable cells in each well were quantified by using the Luna Automated Cell Counter (Logos Biosystems). Cisplatin was kindly provided by Nippon Kayaku (Tokyo, Japan).

### RNA Isolation and qRT-PCR

Total RNA was isolated from P-HEp3, Lu-HEp3, and BM-HEp3 cells by using the RNeasy Mini Kit (Qiagen, Venlo, Netherlands) and was reverse transcribed to cDNA by using the ExScript RT reagent kit (Takara Bio Inc., Otsu, Japan), according to the manufacturers' instructions. The LightCycler System (Roche Diagnostics, Basel, Switzerland) with SYBR Premix DimerEraser (Takara Bio Inc.) was used to perform all PCR reactions. Primers used for qRT-PCR were as follows: TGF-β1 forward: 5′-GTTCAAGCAGAGTACACACAGC-3′; TGF-β1 reverse: 5′-GTATTTCTGGTACAGCTCCACG-3′; TGF-β2 forward: 5′-ATCCCGCCCACTTTCTACAGAC-3′; TGF-β2 reverse: 5′-CATCCAAAGCACGCTTCTTCC-3′; TGF-β3 forward: 5′-TACTATGCCAACTTCTGCTC-3′; TGF-β3 reverse: 5′-AACTTACCATCCCTTTCCTC-3′; CXCR4 forward: 5′-CCCTCCTGCTGACTATTCCC-3′; CXCR4 reverse: 5′-TAAGGCCAACCATGATGTGC-3′; SDF-1 forward: 5′-ACTGGGTTTGTGATTGCCTCTGAAG-3′; SDF-1 reverse: 5′-GGAACCTGAACCCCTGCTGTG-3′; 18S rRNA forward: 5′-CGGCTACCACATCCAAGGAA-3′; and 18S rRNA reverse: 5′-GCTGGAATTACCGCGGCT-3′. Primers were purchased from Sigma. 18S rRNA was used as the internal control.

### Protein Extraction and Immunoblotting

P-HEp3, Lu-HEp3, and BM-HEp3 cells were washed once in ice-cold PBS and then lysed by the addition of CelLytic M Cell Lysis/Extraction Reagent (Sigma) containing freshly added protease inhibitor cocktail (Sigma), 50 mM NaF and 1 mM Na_3_VO_4_. Supernatants were stored at −80 °C until use. The protein concentration was determined using a BCA kit (Pierce Chemical, Rockford, IL). Equal amounts of protein were fractionated via SDS–PAGE and transferred to nitrocellulose membranes (GE Healthcare, Little Chalfont, UK). Membranes were blocked with 5% non-fat dried milk and 0.1% Tween 20 (Sigma) in PBS (pH 7.4) and were then incubated overnight at 4 °C with antibodies against CXCR4 (1: 500; Abcam) and β-actin (1: 5,000; Sigma) in 5% BSA (Sigma) and 0.1% Tween 20 in PBS (pH 7.4). After the membranes were washed, they were incubated for 1 hour in horseradish peroxidase-conjugated secondary antibodies. After a washing, specific protein bands were detected by using ECL Prime Western Blotting Detection Reagents (Amersham Life Science, Arlington Heights, IL) according to the manufacturer's instructions.

### Statistical Analysis

Student's *t* test was used to evaluate differences between two groups. Overall survival of mice was calculated according to the Kaplan-Meier method and was verified by means of the log-rank test. All analyses were performed with JMP software Version 5.1 for Windows (SAS Institute Japan, Tokyo, Japan). Statistical significance was defined as *P* < .05.

## SUPPLEMENTARY MATERIAL AND FIGURES



## References

[1] Aguirre-Ghiso JA (2007). Models, mechanisms and clinical evidence for cancer dormancy. Nat Rev Cancer.

[2] Klein CA (2010). Framework models of tumor dormancy from patient-derived observations. Curr Opin Genet Dev.

[3] Partridge M, Brakenhoff R, Phillips E, Ali K, Francis R, Hooper R, Lavery K, Brown A, Langdon J (2003). Detection of rare disseminated tumor cells identifies head and neck cancer patients at risk of treatment failure. Clin Cancer Res.

[4] Gath HJ, Brakenhoff RH (1999). Minimal residual disease in head and neck cancer. Cancer Metastasis Rev.

[5] Sosa MS, Bragado P, Aguirre-Ghiso JA (2014). Mechanisms of disseminated cancer cell dormancy: an awakening field. Nat Rev Cancer.

[6] Pantel K, Brakenhoff RH, Brandt B (2008). Detection, clinical relevance and specific biological properties of disseminating tumour cells. Nat Rev Cancer.

[7] Meads MB, Hazlehurst LA, Dalton WS (2008). The bone marrow microenvironment as a tumor sanctuary and contributor to drug resistance. Clin Cancer Res.

[8] Polzer B, Klein CA (2013). Metastasis awakening: the challenges of targeting minimal residual cancer. Nat Med.

[9] Schewe DM, Aguirre-Ghiso JA (2008). ATF6α-Rheb-mTOR signaling promotes survival of dormant tumor cells *in vivo*. Proc Natl Acad Sci U S A.

[10] Zhang XH, Wang Q, Gerald W, Hudis CA, Norton L, Smid M, Foekens JA, Massagué J (2009). Latent bone metastasis in breast cancer tied to Src-dependent survival signals. Cancer Cell.

[11] Malanchi I, Santamaria-Martínez A, Susanto E, Peng H, Lehr HA, Delaloye JF, Huelsken J (2012). Interactions between cancer stem cells and their niche govern metastatic colonization. Nature.

[12] Kang Y, Siegel PM, Shu W, Drobnjak M, Kakonen SM, Cordón-Cardo C, Guise TA, Massagué J (2003). A multigenic program mediating breast cancer metastasis to bone. Cancer Cell.

[13] Minn AJ, Gupta GP, Siegel PM, Bos PD, Shu W, Giri DD, Viale A, Olshen AB, Gerald WL, Massagué J (2005). Genes that mediate breast cancer metastasis to lung. Nature.

[14] van ‘t Veer LJ, Dai H, van de Vijver MJ, He YD, Hart AA, Mao M, Peterse HL, van der Kooy K, Marton MJ, Witteveen AT, Schreiber GJ, Kerkhoven RM, Roberts C, Linsley PS (2002). Gene expression profiling predicts clinical outcome of breast cancer. Nature.

[15] Weigelt B, Glas AM, Wessels LF, Witteveen AT, Peterse JL, van't Veer LJ (2003). Gene expression profiles of primary breast tumors maintained in distant metastases. Proc Natl Acad Sci U S A.

[16] Clark EA, Golub TR, Lander ES, Hynes RO (2000). Genomic analysis of metastasis reveals an essential role for RhoC. Nature.

[17] Fidler IJ (1973). Selection of successive tumour lines for metastasis. Nat New Biol.

[18] Bragado P, Estrada Y, Parikh F, Krause S, Capobianco C, Farina HG, Schewe DM, Aguirre-Ghiso JA (2013). TGF-β2 dictates disseminated tumour cell fate in target organs through TGF-β-RIII and p38α/β signalling. Nat Cell Biol.

[19] Ossowski L, Russo H, Gartner M, Wilson EL (1987). Growth of a human carcinoma (HEp3) in nude mice: rapid and efficient metastasis. J Cell Physiol.

[20] Aguirre-Ghiso JA, Ossowski L, Rosenbaum SK (2004). Green fluorescent protein tagging of extracellular signal-regulated kinase and p38 pathways reveals novel dynamics of pathway activation during primary and metastatic growth. Cancer Res.

[21] Hüsemann Y, Geigl JB, Schubert F, Musiani P, Meyer M, Burghart E, Forni G, Eils R, Fehm T, Riethmüller G, Klein CA (2008). Systemic spread is an early step in breast cancer. Cancer Cell.

[22] Jamieson ER, Lippard SJ (1999). Structure, recognition, and processing of cisplatin-DNA adducts. Chem Rev.

[23] Borst P (2012). Cancer drug pan-resistance: pumps, cancer stem cells, quiescence, epithelial to mesenchymal transition, blocked cell death pathways, persisters or what?. Open Biol.

[24] Maeshima AM, Niki T, Maeshima A, Yamada T, Kondo H, Matsuno Y (2002). Modified scar grade: a prognostic indicator in small peripheral lung adenocarcinoma. Cancer.

[25] Yamazaki S, Iwama A, Takayanagi S, Eto K, Ema H, Nakauchi H (2009). TGF-β as a candidate bone marrow niche signal to induce hematopoietic stem cell hibernation. Blood.

[26] Padua D, Zhang XH, Wang Q, Nadal C, Gerald WL, Gomis RR, Massagué J (2008). TGF-β primes breast tumors for lung metastasis seeding through angiopoietin-like 4. Cell.

[27] Calon A, Espinet E, Palomo-Ponce S, Tauriello DV, Iglesias M, Céspedes MV, Sevillano M, Nadal C, Jung P, Zhang XH, Byrom D, Riera A, Rossell D, Mangues R (2012). Dependency of colorectal cancer on a TGF-β-driven program in stromal cells for metastasis initiation. Cancer Cell.

[28] Stankic M, Pavlovic S, Chin Y, Brogi E, Padua D, Norton L, Massagué J, Benezra R (2012). TGF-β-Id1 signaling opposes Twist1 and promotes metastatic colonization via a mesenchymal-to-epithelial transition. Cell Rep.

[29] Kojima Y, Acar A, Eaton EN, Mellody KT, Scheel C, Ben-Porath I, Onder TT, Wang ZC, Richardson AL, Weinberg RA, Orimo A (2010). Autocrine TGF-beta and stromal cell-derived factor-1 (SDF-1) signaling drives the evolution of tumor-promoting mammary stromal myofibroblasts. Proc Natl Acad Sci U SA.

[30] Burger JA, Bürkle A (2007). The CXCR4 chemokine receptor in acute and chronic leukaemia: a marrow homing receptor and potential therapeutic target. Br J Haematol.

[31] Al-Hajj M, Wicha MS, Benito-Hernandez A, Morrison SJ, Clarke MF (2003). Prospective identification of tumorigenic breast cancer cells. Proc Natl Acad Sci U S A.

[32] Pantel K, Schlimok G, Braun S, Kutter D, Lindemann F, Schaller G, Funke I, Izbicki JR, Riethmüller G (1993). Differential expression of proliferation-associated molecules in individual micrometastatic carcinoma cells. J Natl Cancer Inst.

[33] Nie Y, Han YC, Zou YR (2008). CXCR4 is required for the quiescence of primitive hematopoietic cells. J Exp Med.

[34] Chu CY, Sheen YS, Cha ST, Hu YF, Tan CT, Chiu HC, Chang CC, Chen MW, Kuo ML, Jee SH (2013). Induction of chemokine receptor CXCR4 expression by transforming growth factor-β1 in human basal cell carcinoma cells. J Dermatol Sci.

[35] Bertran E, Crosas-Molist E, Sancho P, Caja L, Lopez-Luque J, Navarro E, Egea G, Lastra R, Serrano T, Ramos E, Fabregat I (2013). Overactivation of the TGF-β pathway confers a mesenchymal-like phenotype and CXCR4-dependent migratory properties to liver tumor cells. Hepatology.

[36] Zhao XP, Huang YY, Huang Y, Lei P, Peng JL, Wu S, Wang M, Li WH, Zhu HF, Shen GX (2010). Transforming growth factor-β1 upregulates the expression of CXC chemokine receptor 4 (CXCR4) in human breast cancer MCF-7 cells. Acta Pharmacol Sin.

[37] Hannigan A, Smith P, Kalna G, Lo Nigro C, Orange C, O'Brien DI, Shah R, Syed N, Spender LC, Herrera B, Thurlow JK, Lattanzio L, Monteverde M, Maurer ME (2010). Epigenetic downregulation of human disabled homolog 2 switches TGF-β from a tumor suppressor to a tumor promoter. J Clin Invest.

[38] Curnow SJ, Wloka K, Faint JM, Amft N, Cheung CM, Savant V, Lord J, Akbar AN, Buckley CD, Murray PI, Salmon M (2004). Topical glucocorticoid therapy directly induces up-regulation of functional CXCR4 on primed T lymphocytes in the aqueous humor of patients with uveitis. J Immunol.

[39] Chen S, Tuttle DL, Oshier JT, Knot HJ, Streit WJ, Goodenow MM, Harrison JK (2005). Transforming growth factor-β1 increases CXCR4 expression, stromal-derived factor-1α -stimulated signalling and human immunodeficiency virus-1 entry in human monocyte-derived macrophages. Immunology.

[40] Buckley CD, Amft N, Bradfield PF, Pilling D, Ross E, Arenzana-Seisdedos F, Amara A, Curnow SJ, Lord JM, Scheel-Toellner D, Salmon M (2000). Persistent induction of the chemokine receptor CXCR4 by TGF-β1 on synovial T cells contributes to their accumulation within the rheumatoid synovium. J Immunol.

[41] Wendt MK, Allington TM, Schiemann WP (2009). Mechanisms of the epithelial-mesenchymal transition by TGF-β. Future Oncol.

[42] Singh A, Settleman J (2010). EMT, cancer stem cells and drug resistance: an emerging axis of evil in the war on cancer. Oncogene.

[43] Bertran E, Caja L, Navarro E, Sancho P, Mainez J, Murillo MM, Vinyals A, Fabra A, Fabregat I (2009). Role of CXCR4/SDF-1α in the migratory phenotype of hepatoma cells that have undergone epithelial-mesenchymal transition in response to the transforming growth factor-β. Cell Signal.

[44] Sun CK, Chua MS, He J, So SK (2011). Suppression of glypican 3 inhibits growth of hepatocellular carcinoma cells through up-regulation of TGF-β2. Neoplasia.

[45] Giancotti FG (2013). Mechanisms governing metastatic dormancy and reactivation. Cell.

[46] Zhang XH, Jin X, Malladi S, Zou Y, Wen YH, Brogi E, Smid M, Foekens JA, Massagué J (2013). Selection of bone metastasis seeds by mesenchymal signals in the primary tumor stroma. Cell.

[47] Henckaerts E, Langer JC, Orenstein J, Snoeck HW (2004). The positive regulatory effect of TGF-β2 on primitive murine hemopoietic stem and progenitor cells is dependent on age, genetic background, and serum factors. J Immunol.

[48] Ao M, Franco OE, Park D, Raman D, Williams K, Hayward SW (2007). Cross-talk between paracrine-acting cytokine and chemokine pathways promotes malignancy in benign human prostatic epithelium. Cancer Res.

[49] Vanharanta S, Massagué J (2013). Origins of metastatic traits. Cancer Cell.

[50] Fidler IJ (2003). The pathogenesis of cancer metastasis: the ‘seed and soil’ hypothesis revisited. Nat Rev Cancer.

