# Supplementary material for: Intrinsic TGF-β2-triggered SDF-1-CXCR4 signaling axis is crucial for drug resistance and a slow-cycling state in bone marrow-disseminated tumor cells
Source: Oncotarget. 2014 Nov 25;6(2):1008–19. doi: 10.18632/oncotarget.2826 (PMC4359213; doi:10.18632/oncotarget.2826)
Supplement: Supplementary file 1 [file oncotarget-06-1008-s001.pdf]

Intrinsic TGF-β2-triggered SDF-1-CXCR4 signaling axis is crucial for drug resistance and a slow-cycling state in bone marrow-disseminated tumor cells

Supplementary Material

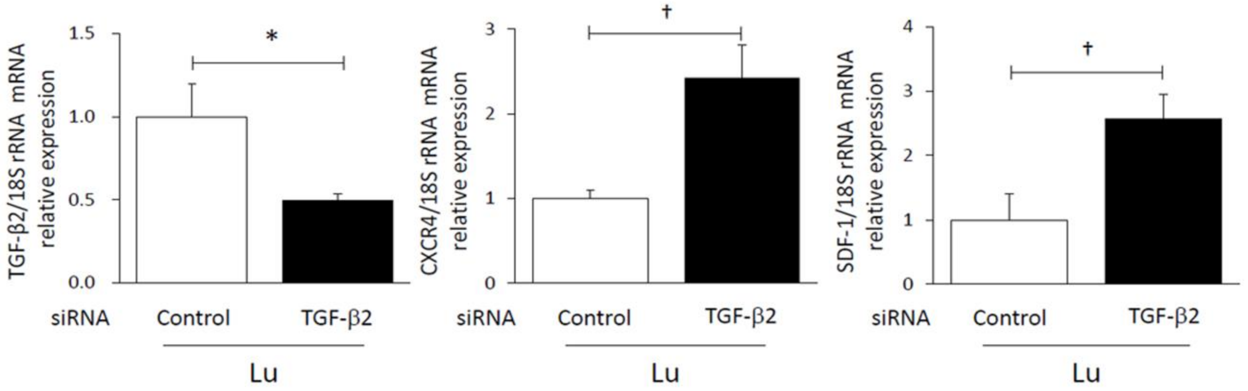

Supplementary Figure S1: Effects of TGF-β2 knockdown on CXCR4 and SDF-1 expression in Lu-HEp3 cells. Expression levels of TGF-β2 (left), CXCR4 (middle), and SDF-1 (right) mRNAs in Lu-HEp3 (Lu) cells was measured by means of qRT-PCR 48 hours after TGF-β2 siRNA transfection. *$P < .05$, $^{\dagger}P < .01$. Values are means ± SEM of triplicate samples.

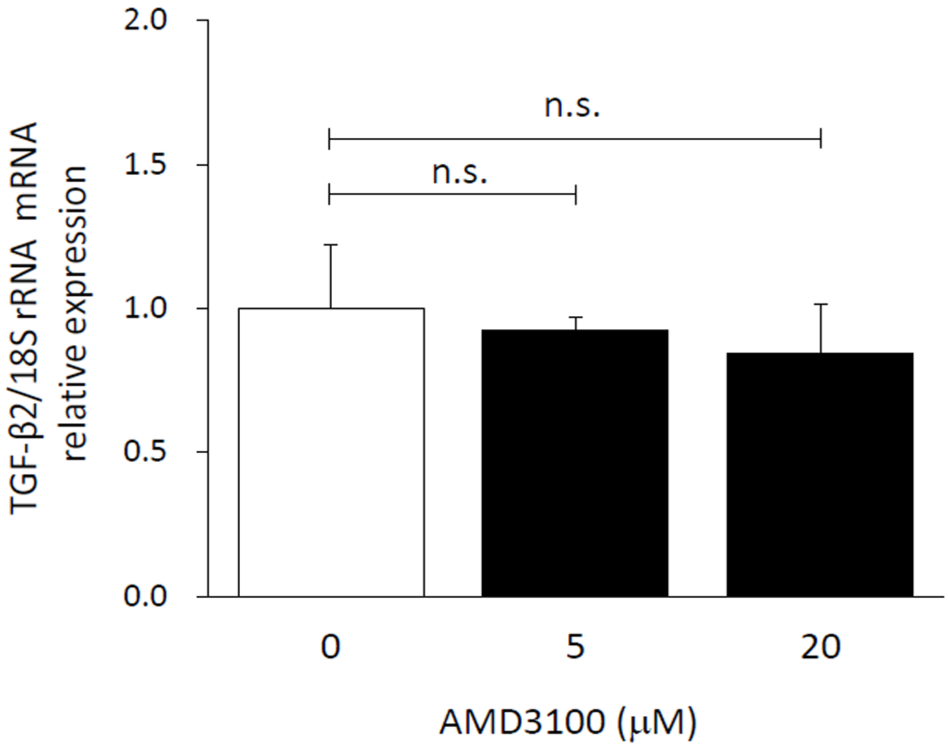

Supplementary Figure S2: Effects of CXCR4 inhibition on TGF-β2 expression in BM-HEp3 cells. TGF-β2 mRNA expression in BM-HEp3 cells treated with AMD3100 at the indicated concentrations for 24 hours was determined via qRT-PCR. n.s., not significant. Values are means ± SEM of triplicate samples.
